# Using a multi-stakeholder experience-based design process to co-develop the Creating Active Schools Framework

**DOI:** 10.1186/s12966-020-0917-z

**Published:** 2020-02-07

**Authors:** Andy Daly-Smith, Thomas Quarmby, Victoria S. J. Archbold, Nicola Corrigan, Dan Wilson, Geir K. Resaland, John B. Bartholomew, Amika Singh, Hege E. Tjomsland, Lauren B. Sherar, Anna Chalkley, Ash C. Routen, Darren Shickle, Daniel D. Bingham, Sally E. Barber, Esther van Sluijs, Stuart J. Fairclough, Jim McKenna

**Affiliations:** 1 School of Sport, Leeds Beckett University, Headingley Campus, Leeds, LS17 7TL UK; 2 Center for Physically Active Learning, Faculty of Education, Arts and Sports, Western Norway University of Applied Sciences, Sogndal, Norway; 3 Born in Bradford, Bradford Institute for Health Research, Bradford Teaching Hospitals Foundation Trust, Bradford, UK; 4 Public Health England (Yorkshire and Humber Centre), London, UK; 5 Yorkshire Sport Foundation, Gildersome, UK; 6 Department of Kinesiology and Health Education, The University of Texas at Austin, Austin, TX USA; 7 Department of Public and Occupational Health, Amsterdam UMC, Amsterdam Public Health, Amsterdam, The Netherlands; 8 Mulier Institute, Utrecht, the Netherlands; 9 School of Sport, Exercise and Health Sciences, Loughborough University, Loughborough, UK; 10 NIHR Applied Research Collaboration East Midlands (ARC EM), Diabetes Research Centre, University of Leicester, Leicester, UK; 11 Leeds Institute of Health Sciences, University of Leeds, Leeds, UK; 12 Centre for Diet and Activity Research, MRC Epidemiology Unit, University of Cambridge, Cambridge, UK; 13 Dept. Sport and Physical Activity, Edge Hill University, Ormskirk, UK

**Keywords:** Whole-school, Children, Whole-system, Double diamond, Co-development, Physical activity, Policy, Physical education, Experience-based co-design

## Abstract

**Background:**

UK and global policies recommend whole-school approaches to improve childrens’ inadequate physical activity (PA) levels. Yet, recent meta-analyses establish current interventions as ineffective due to suboptimal implementation rates and poor sustainability. To create effective interventions, which recognise schools as complex adaptive sub-systems, multi-stakeholder input is necessary. Further, to ensure ‘systems’ change, a framework is required that identifies all components of a whole-school PA approach. The study’s aim was to co-develop a whole-school PA framework using the double diamond design approach (DDDA).

**Methodology:**

Fifty stakeholders engaged in a six-phase DDDA workshop undertaking tasks within same stakeholder (*n* = 9; UK researchers, public health specialists, active schools coordinators, headteachers, teachers, active partner schools specialists, national organisations, Sport England local delivery pilot representatives and international researchers) and mixed (*n* = 6) stakeholder groupings. Six draft frameworks were created before stakeholders voted for one ‘initial’ framework. Next, stakeholders reviewed the ‘initial’ framework, proposing modifications. Following the workshop, stakeholders voted on eight modifications using an online questionnaire.

**Results:**

Following voting, the Creating Active Schools Framework (CAS) was designed. At the centre, ethos and practice drive school policy and vision, creating the physical and social environments in which five key stakeholder groups operate to deliver PA through seven opportunities both within and beyond school. At the top of the model, initial and in-service teacher training foster teachers’ capability, opportunity and motivation (COM-B) to deliver whole-school PA. National policy and organisations drive top-down initiatives that support or hinder whole-school PA.

**Summary:**

To the authors’ knowledge, this is the first time practitioners, policymakers and researchers have co-designed a whole-school PA framework from initial conception. The novelty of CAS resides in identifying the multitude of interconnecting components of a whole-school adaptive sub-system; exposing the complexity required to create systems change. The framework can be used to shape future policy, research and practice to embed sustainable PA interventions within schools. To enact such change, CAS presents a potential paradigm shift, providing a map and method to guide future co-production by multiple experts of PA initiatives ‘with’ schools, while abandoning outdated traditional approaches of implementing interventions ‘on’ schools.

## Background

Globally, 50% of children do not meet the internationally recognised target of 60 min of moderate-to-vigorous physical activity (MVPA) per day [1, 2]. This figure rises to 80% in higher-income countries [2] and persists into adolescence [3]. Given these figures, it is not surprising that the latest global physical activity report card states “children’s physical activity poses a serious level of concern” [4]. To effectively address such high levels of concern, health promotion endeavours need to target all levels and settings. As the majority of children have exposure to school it is unsurprising that global and UK policy recommend whole-school approaches as one of the most promising investments for physical activity in childhood [5–8]. Specifically, within the UK, the government provide ring-fenced funding to support primary schools (children aged 5 to 11) to provide a minimum of 30-min of physical activity per day for all pupils [9, 10].

Despite these calls, it remains unclear what are the most effective whole-school approaches to sustain change and how they can be successfully implemented. Concurrently, recent meta-analyses’ establish that school-based interventions have little, if any, effect on school-time MVPA [11] or daily MVPA [12]. This may result from the challenge of designing and delivering feasible and sustainable approaches in schools, as evidenced by the many randomised controlled trials of school-based physical activity programmes displaying poor implementation e.g. [13–15]. These findings have been attributed to ‘top-down’ approaches where researchers and external stakeholders drive intervention design with limited input from school stakeholders [16]. Co-production of interventions by all stakeholders is therefore essential, galvanising both bottom-up and top-down approaches to create ‘systems change’ [5, 17].

The failure to establish effective physical activity interventions suggests a need to mobilise schools and align the willing support being offered by associated stakeholders. To do so, a whole-school physical activity framework is required that moves beyond the conceptual understanding of the school environment to one which presents schools as a wider ‘complex adaptive sub-system’ [18, 19]. Complex adaptive systems possess “many heterogeneous components that dynamically interact and produce an emergent effect greater than the individual elements, which must persist and adapt to changing circumstances” [20]. We believe the deficiency of current conceptual models to present a map of the many component parts likely explains why schools and associated stakeholders fail to implement interventions at the desired level and sustain implementation over an extended period of time [21–24]. Further, while all frameworks (e.g. comprehensive school physical activity programme [21]) incorporate socio-ecological theory, no frameworks have embedded a modern understanding of behavioural science (e.g. COM-B) [25]. This is essential to enable all stakeholders to appreciate the magnitude of the factors that need to be addressed and, adopt evidence-based approaches to change the behaviours and create system change.

New integrated approaches have emerged that enable the identification and combination of the expertise of multiple stakeholders in the development of systems approaches [16]. Yet, to date, these have not been applied within the school setting, nor to design a comprehensive whole-school physical activity framework. Importantly, these human-centered approaches focus on outcomes that enthuse, incentivise, and build on the strengths of all stakeholders [26]. One common approach - experience-based co-design - has been widely used to create systems change in emergency medicine and mental health care settings [27, 28]. A specific method of experience-based co-design is the *Double Diamond Design Approach* (DDDA) [29] that has been used to develop service improvements in health and social care [30], patient-centered cancer treatment facilities [31], and organisational medical care [32]. The DDDA draws on recent discoveries on how to optimise both divergent - creating choices- and convergent - making choices - creative thinking processes [29, 33]. With DDDA, stakeholders progress through a four-stage reflective process to discover, define, develop, and deliver an innovative solution to a problem. The strength of this design approach resides in the collaboration between multiple stakeholders within an innovative development process to produce an understanding greater than the sum of the individual parts [26].

The aim of the current study was to co-develop a whole-school physical activity framework with multiple stakeholders, using the DDDA. To our knowledge, this will be the first UK-based whole-school physical activity framework and the first time that any framework has involved experience-based co-design from conception. Given the novelty of this approach, the following section will present a detailed methodology of the design approach to demonstrate the iterative nature of the design process as each phase impacts the next.

## Methodology: framework development process

### Participants

Purposive sampling [28], a key facet of experience-based co-design methodology, was used to identify participants for the six initial same stakeholder groups (1. UK researcher, 2. public health specialist, 3. active schools coordinator, 4. headteacher, 5. teacher and 6. active partner schools specialist; stakeholder descriptors, see Additional file 1). Participants (*n* = 50, Table 1) were recruited through networks of three of the authors (research, ADS; practice, DW; policy, NC). Other than national researchers and public health representatives, all participants were recruited from across the Yorkshire and Humber region, one of the largest regions within the UK (5.4 million people across 15 local authorities; 1776 primary schools). The region has the same levels of physical (in) activity and educational outcomes than the rest of the UK, resulting from a broad range of ethnicities, socioeconomic status and rural/urban landscapes. The stakeholders were specifically compiled to ensure representation of the different demographics, local authorities and a range of professional experiences. To ensure a broad national perspective (i.e. to include opportunities and barriers beyond the experience of regional schools), we invited national organisations and representatives for the Sport England Local Delivery Pilots. In addition, active schools researchers from beyond the UK were invited to present an international perspective. No limits were placed on the number of participants in the final three stakeholder groups. Participants were contacted via telephone and/or e-mail and were required to return consent prior to engaging in the workshop. Ethical clearance was granted by Leeds Beckett University Research Ethics Committee (N^o^ 60271).

**Table 1:** Key stakeholder characteristics

Stakeholder Group	Proportion (*%*) of males/females	Years in current role *mean (range)*	Years in current profession *mean (range)*	Years as a qualified teacher *mean (range)*
UK researchers (*n* = 6)	33/67	6.46 (3.0–12.0)	13.50 (10.0–18.0)	–
Public Health specialists (*n* = 5)	0/100	2.40 (0.8–3.0)	15.30 (8.0–21.5)	–
Active school coordinators (*n* = 6)	17/83	8.17 (2.0–16.0)	13.96 (7.8–20.0)	17.75 (0.0–32.0)
Headteachers (*n* = 6)	83/17	6.20 (3.8–9.8)	18.47 (13.7–30.0)	18.47 (13.7–30.0)
Teachers (*n* = 6)	50/50	4.93 (0.7–11.7)	13.38 (7.0–20.0)	11.71 (0.0–20.0)
Active partner school specialists (*n* = 6)	33/67	3.63 (0.4–9.8)	6.04 (0.4–15.0)	2.67 (0.0–16.0)
National organisation representative(*n* = 5)	40/60	5.9 (1.17–16.0)	20.0 (10.0–41.0)	11.8 (0.0–41.0)
Local deliver pilot representatives (*n* = 5)	80/20	5.43 (0.3–15.0)	20.8 (10.0–40.0)	15.0 (0.0–40.0)
International researchers (*n* = 5)	60/40	11.62 (1.0–22.0)	18.73 (5.0–28.7)	0.40 (0.0–2.0)

### Overview

**Fig. 1:**
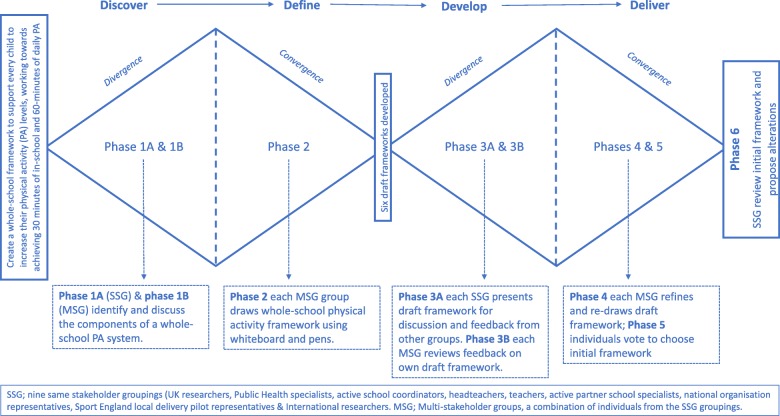
The Double Diamond Design Approach used to develop the Creating Active Schools Framework

The initial whole-school physical activity framework development phase took place during a two-day event in June 2019, Leeds, UK. On day one, the stakeholders observed a conference for 80 school senior leaders and governors on whole-school approaches to physical activity. Specifically, the six initial stakeholder groups were tasked with observing an idea generation workshop where school leaders were tasked with identifying strategies to increase MVPA by at least two minutes during one of seven selected school-day segments. A two-minute increase was suggested to encourage school leaders to identify small changes that were more feasible to implement in a packed curricular and were, therefore, more likely to be sustainable. On day two, the stakeholders engaged in the whole-school physical activity framework development process using the DDDA [29] (stage 1; Fig. 1). Following the framework development day, an online questionnaire (stage 2) was used to modify the ‘initial’ whole-school physical activity framework developed on day two.

### Stage one: double diamond design approach

The DDDA was divided into six phases with one or more tasks (A/B) per phase (outline; Fig. 1). Within each phase, stakeholders worked in the same stakeholder groups (e.g. teachers) or mixed stakeholder groups. All discussions were recorded via dictaphones on each table. Stakeholders were allocated to mixed stakeholder groups using the following principles: first, one member from each of the six initial same stakeholders groups, ensuring a balance in the number of years of experience. Second, stakeholders from the national organisations, local delivery pilots and international researchers were allocated to a group, ensuring a balance in experience and group numbers. Due to illness, only five Public Health Specialists were present. Therefore, stakeholders from the local delivery pilot were allocated first and were asked to play dual roles where they possessed the relevant expertise.

The workshop began with a summary of day one. Next, lead researchers briefed participants on the DDDA and the expected outcome, which was to *create a whole-school physical activity framework that would support every child to increase their physical activity levels, working towards achieving 30 min of in-school and 60-min of daily MVPA*. Stakeholders were not introduced to examples of current school-based physical activity frameworks [21, 23, 34] until the beginning of phase four. The research leads took this decision as they did not want to influence the initial designs. In addition, the UK and international researchers were members of each mixed stakeholder group and were aware of the different frameworks so could refer to these within group discussions if it was deemed appropriate.

The six development phases are outlined in Fig. 1. Specifically, within phase five, individuals voted for the draft framework that best achieved the brief. Each participant received three votes with a maximum of two votes being allowed for any one framework. Any uncast votes were placed on the side of the voting slip. Participants were not allowed to vote for their own framework. Overall, mixed stakeholder group four’s draft framework received the most votes (average number of votes per individual 1.2, see Additional file 2). When the votes were broken down by the same stakeholder groups, framework four was the most popular choice in seven of the nine groupings.

Within phase six, the participants reformed into same stakeholder groups to review framework four, the “initial framework” discussing; i) what was good about the ‘initial’ framework and what needed improving? ii) how the framework may be used by their stakeholder group and iii) the next steps? On completion of phase six, the lead facilitators drew the workshop to a close and informed the participants of the next whole-school physical activity framework development stage.

### Stage two: online questionnaire

An additional stage - beyond the DDDA - was undertaken to refine the ‘initial’ framework (framework four) to ensure it reflected the needs of all stakeholders. The refinement process was undertaken remotely via an online questionnaire. To develop the questionnaire, the lead author extracted proposed modifications to the ‘intial’ framework from the audio recordings from the nine same stakeholder groups phase six, final discussions. Eight proposed modifications were suggested (Table 2), with visual modifications being made to framework four to demonstrate each proposed alteration. Finally, the prototype frameworks that represented each proposed modification were viewed, discussed and approved by four authors. Once approved, an online questionnaire was created to enable the original stakeholders to vote (sequentially) on the suggested modifications. This was emailed two weeks after the initial workshop and remained open for two weeks.

**Table 2:** The proportion (%) of stakeholders who agreed with the eight proposals to modify the initial whole-school physical activity framework

	1. Change skills, knowledge & competence to capability, opportunity & motivation	2. Change teacher practice & ethos to whole-school practice & ethos.	3. Change 5 original pillars to 5 people- orientated pillars	4. Show social & physical environment as interweaving through the 5 pillars.	5. Present the five pillars and social/ physical environments as a DNA helix?	6. Introduce a new part to the model where children are included as the main beneficiaries?	7. Change the best-practice physical activity box to include 7 PA segments/ opportunities?	8. Rotate the model 90 degrees to the left?
UK Researchers (n = 5)	100	100	80	100	20	80	60	40
Public Health (n = 5)	100	80	100	100	0	80	80	80
Active Schools (*n* = 4)	75	100	100	100	50	100	50	50
Head Teachers (n = 5)	100	100	100	80	60	80	40	60
Teachers (n = 5)	100	80	60	100	60	100	60	20
Active Partners (n = 4)	100	100	100	75	25	100	75	50
Nat Organisations (*n* = 3)	33	67	100	100	67	100	100	100
LDP (*n* = 1)	100	100	100	100	100	100	100	100
Int Researchers (n = 5)	100	100	100	80	40	80	0	40
Total (*n* = 37)								
Overall in agreement	**92**	**92**	**92**	**92**	41	**89**	**57**	54
Number of groups with +ve response	**8 of 9**	**9 of 9**	**9 of 9**	**9 of 9**	4 of 9	**9 of 9**	**6 of 9**	4 of 9
Accept/ Decline modification	**Accept**	**Accept**	**Accept**	**Accept**	Decline	**Accept**	**Accept**	Decline

Modifications were accepted when > 50% of the total sample voted for the change and more than half of the stakeholder groups (5 or more) voted for the change. **Bold text** indicates proposal acceptance

## Results

### Online questionnaire results: modification to the “initial framework”

Seventy-four percent of the original stakeholders responded to the online questionnaire. Proposed modifications were accepted when; > 50% of the participants voted to accept a modification and > 50% of the stakeholder groups (five or more) voted for the modification. Proposals one, two, three, four, six and seven were accepted (Table 2). Proposals five and eight were rejected. Finally, due to proposal five being declined, four authors designed an alternate representation of the social and physical environment, weaving the environments through the five people-orientated pillars.

### Creating Active Schools Framework: description

#### Overview

**Fig. 2:**
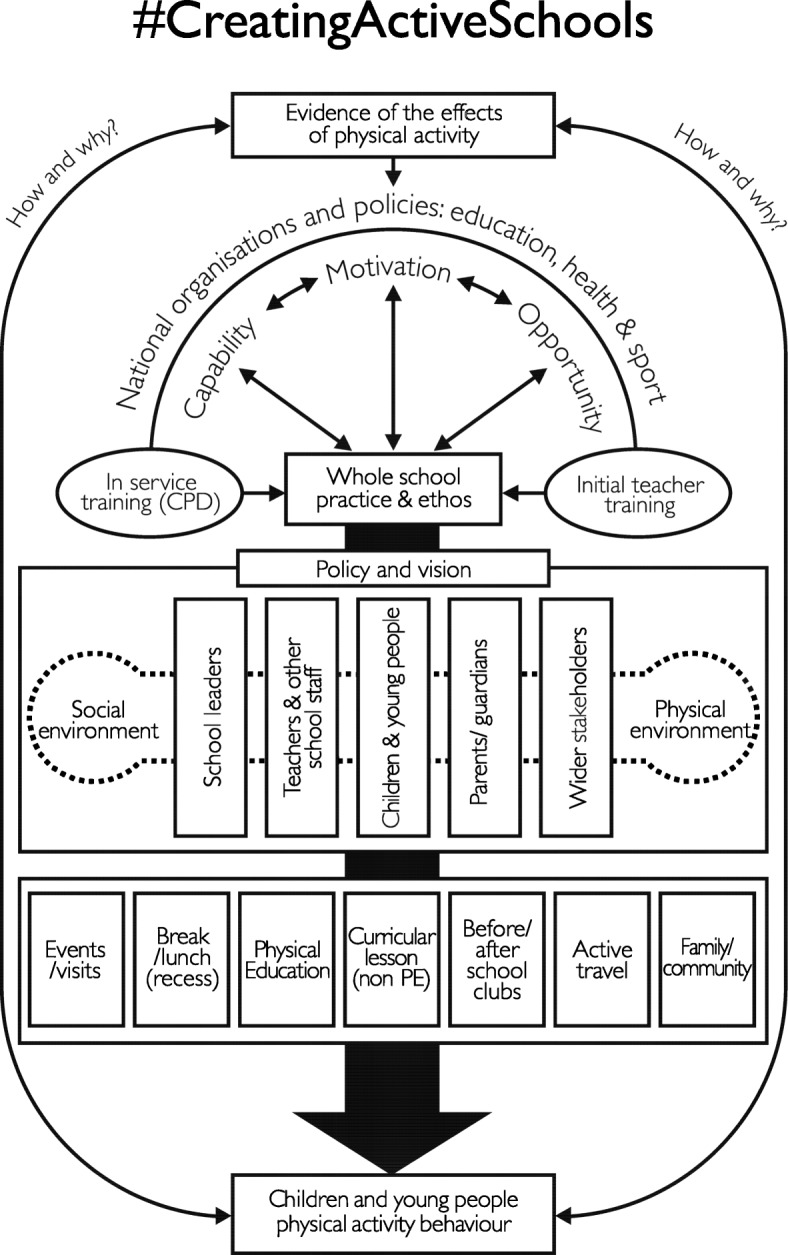
Creating Active Schools (CAS) Framework

The primary outcome of the study was the CAS Framework (Fig. 2). The framework identifies the multiple components required to establish schools as a complex adaptive sub-system which, in turn, will facilitate whole-school physical activity implementation. The bottom half of the framework outlines the in-school factors, while the top half identifies factors associated with teacher training, behavioural science and the role of national and international organisations and policy development; the wider system beyond an individual school.

#### Whole school practice and ethos

The cornerstone of the CAS framework is establishing whole-school practice and ethos for physical activity - the underlying sentiment that informs the beliefs, customs and practices around creating a physically active school; the central box. Working downwards, whole-school practice and ethos drives internal school policy and vision, both essential components of creating a whole-school physical activity approach through engaging relevant stakeholders and creating facilitative social and physical environments [35, 36].

#### Key stakeholders

Five groups are included in policy and vision as essential stakeholders; school leaders, teachers and other school staff, children/young people, parents/guardians, and wider stakeholders (e.g. active school coordinators, public health specialists). School leaders (principal, wider senior leadership team and governors) are responsible for leading the development of the policy and vision statements and managing associated resources. Teachers and wider school staff are central to creating positive social and physical environments, alongside delivering initiatives within the seven opportunities. Other school staff include playground supervisors or teaching assistants, both of whom can play an important role in whole-school physical activity provision. Children and young people may form pupil councils or lead opportunities for physical activity (e.g. playground leaders). Parents/ guardians play a significant role in supporting children to engage in extracurricular activities and may also form parent associations. Wider stakeholders may include; active school coordinators, active partner school specialists or external private, charity or voluntary sector organisations who deliver initiatives within the seven opportunities, or, support schools with systems-level change. All stakeholders are essential to creating and sustaining a whole-school physical activity approach.

#### The social and physical environment

The five people-orientated pillars operate within the physical and social environment. The physical environment reflects the amount, variety (e.g. green space, playground, school hall) and quality of school spaces and resources available [37]. The social environment reflects the degree to which the stakeholders engage and support each other to provide physical activity. For example, teachers who implement physically active learning within supportive school social environments experience fewer implementation barriers [38–40].

#### Seven opportunities for physical activity

Combined, the environment and key stakeholders determine the implementation of physical activity across seven opportunities. The opportunities are determined by what the school can control (from the centre to the left) and opportunities that the school can influence (to the right of centre). The opportunities with the greatest potential impact on whole-day physical activity reside closest to the framework midline. Expanding physical activity into curricular lessons (not Physical Education) using exercise breaks or physically active learning both enhance levels of MVPA [41, 42]. Moving left, Physical Education [43–46] and break/ lunch (recess) [46–48] interventions that extend the duration, increase the frequency and/or enhance the delivery have been shown to be effective [49]. Finally, trips (e.g. museums) and events (e.g. sports day, summer fair) provide one-off opportunities for physical activity engagement.

Right of centre, before/ after school clubs and active travel are opportunities that schools can influence but cannot control, as responsibility largely resides with children and their families. Once engaged, the school does, however, play a central role in determining the amount and quality of such provision (e.g. active travel plans). If successful, both opportunities can significantly contribute to a child’s whole-day physical activity levels [11, 50–53]. Finally, the school can influence family and community physical activity beyond school time. This may involve providing active homework [54] or opening school facilities beyond the school day to support community organisations [55]. The culmination of establishing a whole-school practice and ethos that includes physical activity focused policies and vision, positive environments, engagement with stakeholders and provision of effective opportunities, will enhance the amount and quality of physical activity experiences children receive in and beyond school.

#### Teacher training and behavioural change theory

Working upward from whole-school practice and ethos, wider political and policy systems - beyond the school - strongly influence the in-school provision of physical activity. Initial teacher training and in-service training (CPD) are central to enhancing the capability and motivation of school staff to implement physical activity. Currently, initial teacher training fails to provide newly qualified teachers with sufficient capability to become effective whole-school physical activity practitioners [34, 56–58]. Until initial teacher training evolves to meet the demands of contemporary teachers who are tasked with delivering a curriculum focussed on physical, social and emotional development, high-quality in-service training (CPD) is required [58]. Training should enhance delivery skills, while also upskilling teachers and school leaders to lead systems change for physical activity. Behavioural science is a required component of all training programmes (initial teacher training or in-service training (CPD)) to enhance the capability, opportunity and motivation (COM-B, Michie et al., 2011) of all stakeholders and the school system, thus maximising the likelihood of change. This, in turn, will influence the capability, motivation and opportunity of the children within the schools.

#### National organisations and policy

The final part of the framework involves national organisations and policies that drive the educational focus of schools and the training needs of the key stakeholders. While physical activity may not be front and centre of such policies, it is essential that they align to support physical activity and avoid inadvertently promoting conflicting behaviours (e.g. prolonged sitting in lessons). Finally, it is essential that the framework - and approaches within - are informed by research evidence to ensure the highest quality provision for the children whom they serve.

## Discussion

To the authors’ knowledge, this is the first time that practitioners, policymakers and researchers who understand the powerful driving agents of school systems and teacher and pupil physical activity behaviour, have co-designed a whole-school physical activity framework from initial conception. The underlying DDDA, previously used to develop high-quality systems change in multiple healthcare settings, was used to develop and innovative practice and evidence-based framework that meets the needs of each stakeholder as well as the important constituent parts required for effective and sustainable implementation. To achieve such an outcome, it was essential for the design process to incorporate multiple stages of divergent and convergent thinking to optimise the final framework by challenging and refining initial views. As a result, CAS has high face validity, and because it has been endorsed by a range of professional groups, it also demonstrates professional and contextual credibility.

### Implications for practice

Providing greater detail than previous frameworks [21–24], CAS confirms the large number of components that must be addressed so schools can evolve into adaptive sub-systems with physical activity at the heart of their provision. While little is currently known about the inter-relationship between the different elements - and CAS does not identify those with the greatest effect - it is the first framework to establish whole-school ethos and practice at the heart of whole-school physical activity provision. Importantly, and in agreement with previous frameworks, CAS refutes the notion of deploying single-element interventions; it reinforces the need to create systems change through school leadership groups. Such change has already been identified within current research and frameworks [21, 24], yet remains elusive within the school environment [34, 59].

Framing every school as a unique, complex adaptive sub-system, CAS establishes the importance of whole-school ethos and practice. This is consistent with Meadow’s [60] 12 levers of influence where value-based leverage is central to creating sustainable change; this is best achieved by (i) identifying systems goals, (ii) understanding the paradigm guiding the design of the new system and (iii) encouraging a shift in the decision-making paradigm as new challenges arise. The dynamism and complexity outlined by the potential interplay of so many facets helps to put flesh on the bones of the notion of ‘compensating feedback’, which explains why powerful sub-systems, like schools, resist powerfully [61]. To create change, CAS confirms that so much needs to be made right. This was most evident in the interactions between the five stakeholders within the social and physical environments; these are levels that many physical activity initiatives and previous frameworks overlook [5].

The CAS framework presents seven physical activity opportunities, a greater number than observed in previous frameworks [21]. The specific positioning of each opportunity draws on contemporary physical activity promotion theory (expand, extend and enhance) and a recent meta-analysis to place the most effective toward the framework midline [11, 49]. Combined, this ensures that practitioners are being guided by the current evidence base to operationalise the most effective physical opportunities.

Of great surprise, the seven opportunities were positioned at the lowest level, suggesting not only their fragility but also the importance of higher-level factors. While previous frameworks and contemporary research recognise the need for higher-level engagement, this is contrary to current practice which is often characterised by limited engagement with different levels of the school system [62]. Consistent with previous whole-school frameworks [21, 24], the CAS framework reminds all stakeholders to move beyond simple interventions to become ‘systems thinkers in action’ [17]; this reminds practitioners to address at least three levels of their school system; the grand system (e.g., Schools), local system (e.g., a single school) and system parts (the mechanisms of individual events/provision) [63].

The old counselling adage of ‘the map is not the territory’ applies to CAS; CAS does not identify the specifics of any individual school. Change leaders who may act as whole-school physical activity champions - as seen in the comprehensive school physical activity programme and Action Schools BC frameworks [21, 24, 64] – can use the framework to develop a bespoke process to fit the unique requirements of their school. With over 20 active components, physical activity champions will require a system change plan. That plan will identify the priorities and modify the existing structures to create change. Perhaps this is the first framework where all of the components have been collated. For the first time, CAS reveals the complex challenges facing these champions, especially primary school teachers and senior leaders with little expertise in physical education delivery, let alone systems change for whole-school physical activity [23]. Given this complexity, as highlighted in the CSPAP partnership framework [23], it makes sense to address these issues in initial teacher training programs and in-service training (CPD); in-service teachers will need to embed the skills that establish (i) the capability, opportunity and motivation for systems change and (ii) systems change that secures whole-school physical activity. Importantly, the CAS framework is the first that embeds a modern, eclectic behaviour change framework (COM-B) [25]. The integration of the COM-B framework reflects a need for accessible language while retaining an underlying complexity of twenty-first-century behavioural decision-making.

Unique to CAS is the practical implementation of the framework where schools and wider stakeholders can promote self-reflection by mapping current provision and identifying underserved components. Maintaining the co-production process, initiatives should be implemented with, rather than on schools. At this stage, it is essential that children, who were not engaged in our co-development process, become equal partners in identifying, developing and implementing future interventions. To support schools, an evidence-based audit tool would emphasise the importance of all CAS framework components, especially whole-school ethos, practice, policy and vision; components often neglected in previous interventions. While CAS was developed within a specific UK context, its flexible nature allows replication elsewhere. Moreover, while secondary (high) schools may benefit from using CAS as a guiding framework, it is important that they establish face validity and acceptability as initial priorities. Perhaps the first step is to identify early adopters and seek to test and learn new/novel processes for creating whole-school practice and ethos aligned to physical activity.

### Implications for policy

Similar to the CSPAP framework, CAS establishes the important role of national organisations and policy. The uniqueness of CAS resides in the graphical representation of the national organisations and policy layer as it reinforces the importance of creating both horizontal and vertical alignment of people, organisations and policies; this will help ensure that all changes move in the same direction. Vertical alignment reflects the need for key issues to be reinforced throughout all processes down to the level of individual pupils and moments within the school day. In contrast, horizontal alignment requires a common shared vision within each level of the system (e.g. national organisations and government departments, e.g., in the UK of Health & Social Care, Education and Digital, Culture, Media & Sport). Misalignment between horizontal and/or vertical issues is likely to create unhelpful friction that challenges the creation of a clear whole-school ethos [17], weakening any resulting interventions. To enact policy and evolve current practice, uniquely, CAS not only provides a checklist for change agents but also a template for the development of a healthy schools rating scheme. Multiple schemes currently exist [65, 66], yet few reflect the full range of influential components. As a result, they may lack the detail required to promote effective and sustainable physical activity initiatives.

Government education, health and sports departments that value physical activity can use the CAS framework to drive strategic change within the education system. Naming all departments, for the first time, promotes the use of one central framework for whole-school physical activity as it can be used to drive combined efforts across all UK government departments and policies. Such alignment, as previously stated, is central to creating a sustainable adaptive system that promotes one vision of creating an active school; CAS is unique in graphically representing this opportunity. Furthermore, bodies that hold schools to account for educational standards (e.g., Office for Standards in Education in the UK) can utilise CAS as a tool to support schools to embed physical activity throughout the school day. In addition, national and localised sport and health organisations that set strategies for grassroots sports and health improvement can use CAS to highlight their role in whole-school physical activity. CAS will enable organisations to align their provision and develop more efficient and sustainable practises in schools and their local communities.

### Implications for research

The CAS framework provides researchers with an understanding of the multiple components that need to be addressed to create and evaluate whole-school physical activity interventions. It emphasises the need for researchers to move beyond push approaches and co-develop interventions with multiple stakeholders within the school setting from conception [16]. The challenge for researchers resides in creating programmes that create systems-level change within schools. Unlike previous frameworks, CAS highlights the need to focus on school-level change, the role of each key stakeholder group and the social and physical environments, not just the interventions within the seven opportunities [62]. CAS, therefore, reveals the required components researchers must be aware of when supporting schools in the design, delivery and evaluation of future physical activity interventions. Further, this study provides a template for physical activity researchers in the UK and beyond to adopt experience-based co-design approaches, specifically using the DDDA approach.

### Strength and limitations

A particular strength of the CAS framework is that it is, to the authors’ knowledge, the first to deploy a co-development methodology with multiple stakeholders holding deep and wide experience of UK school systems. While pioneering, the final framework is based on the vision of a specific group of stakeholders and this specific process. Involvement of further stakeholders (children, parents, school nurses etc) or indeed alternate “experts” may have yielded different outcomes. Yet, CAS reflects insights from the UK and select westernised high-income countries meaning it is likely to provide a reasonable reflection of the components of a whole-school physical activity framework within similar countries and education systems. In addition, the flexibility of CAS enables contrasting school systems to prioritise different components with the framework to meet curricular and logistical needs. Children were not included in the development of the CAS Framework due to the focus on systems and processes, rather than implementation. It is envisaged that children, as key stakeholders, will be central to creating a school’s individual implementation plan and will support the implementation of the actions needed to provide effective physical activity opportunities.

This is the primary instance that an experience-based co-design process - the DDDA - has been used within the whole-school physical activity field. Recognising our relative inexperience in design, and notwithstanding that the paper provides a powerful template for future projects, improvements may emerge from a more refined design process. Further, while the framework provides a map, it does little to identify how the respective parts interact, nor does it specify the optimal sequence(s) or interactions that need to take place [67]. Future research and practice collaborations will need to investigate the implementation of the framework.

## Conclusion

The CAS Framework was co-produced from initial conception by multiple experts who understand the powerful driving agents of school systems and teacher and student physical activity behaviour. The novelty of the CAS framework resides in formally identifying the multitude of interconnecting components of a whole-school adaptive sub-system; this exposes the complexity required to create systems change. The iterative design process, involving multiple stakeholders who understand the layers of influence within and beyond schools, has high face validity which may, for the first time, consolidate and direct the efforts of all stakeholders. The CAS framework can be used to shape future policy, research and practice to embed sustainable physical activity interventions within schools. To enact such change, the CAS framework presents a potential paradigm shift, providing a map and method to guide future co-production by multiple experts of initiatives ‘with’ schools, while abandoning outdated traditional approaches of implementing interventions ‘on’ schools. To facilitate this change, a practical toolkit and resources are required to support schools to implement whole-systems change that meets the needs of their individual setting. To maximise reach, the toolkit should be housed on a web portal with face-to-face workshops for those schools and stakeholders who require more specific support.

## Supplementary information

**Additional file 1.Table S1.** Stakeholder role descriptions

**Additional file 2.** Stage 1, phase 5 voting results; mean votes awarded to each draft framework by stakeholder grouping

## Data Availability

Data sharing not applicable to this article as no datasets were generated or analysed during the current study.

## Abbreviations

CAS Framework: Creating Active Schools Framework
DDDA: Double-diamond design approach
MVPA: Moderate-to-vigorous physical activity
PA: Physical activity
UK: United Kingdom
