# Supplementary material for: Using a multi-stakeholder experience-based design process to co-develop the Creating Active Schools Framework
Source: Int J Behav Nutr Phys Act. 2020 Feb 7;17:13. doi: 10.1186/s12966-020-0917-z (PMC7006100; doi:10.1186/s12966-020-0917-z)
Supplement: Supplementary file 1 — Additional file 1.Table S1. Stakeholder role descriptions [file 12966_2020_917_MOESM1_ESM.docx]

Additional file 1: Table 1 Stakeholder role descriptions

| Role | Description |
| --- | --- |
| UK researchers | A researcher with expertise in the evaluation and implementation expertise of school-based physical activity interventions within the UK |
| Public Health specialists | A professional practitioner with knowledge and skills to support population-level efforts of society to develop policy and interventions to prevent ill-health utilising defined public health principles. |
| Active school coordinators | An organisational lead who supports cross-school, Physical Education sport and physical activity, usually covering between 12 and 40 schools. |
| Headteachers (principal) | A school leader with experience of implementing a whole-school physical activity approach within their own school and in some cases supporting and training further schools. |
| Teachers | A teacher with expertise in leading in-school implementation of physical activity, Physical Education and school sport. |
| Active partner school specialists | A community engagement leader with expertise in supporting schools to implement sport and PA programmes within and beyond the school timetable. |
| National organisation representatives | An individual who works within government organisations, charities or the private sector with a remit to improve the education and health of children through sport, physical activity or education. |
| Local delivery pilot representatives | A UK-based role with strategic leadership or management responsibility in one of 12 Sport England funded initiatives to improve community physical activity through systems change. |
| International researchers | An international researcher with evaluation and implementation expertise of school-based PA interventions beyond the UK. |
