# Supplementary material for: Using a multi-stakeholder experience-based design process to co-develop the Creating Active Schools Framework
Source: Int J Behav Nutr Phys Act. 2020 Feb 7;17:13. doi: 10.1186/s12966-020-0917-z (PMC7006100; doi:10.1186/s12966-020-0917-z)
Supplement: Supplementary file 2 — Additional file 2. Stage 1, phase 5 voting results; mean votes awarded to each draft framework by stakeholder grouping [file 12966_2020_917_MOESM2_ESM.docx]

Additional file 2 The mean number of votes awarded to each whole-school physical activity framework per participant within each stakeholder group.

|  | Framework one | Framework two | Framework three | Framework four | Framework five | Framework six | Uncast votes |
| --- | --- | --- | --- | --- | --- | --- | --- |
| UK researchers  (*n*=6) | 0.6 | 0 | 0 | **1.2** | 0.8 | 0 | 0.4 |
| Public Health specialists (*n*=5) | 0.5 | 0.0 | 0.2 | **1.5** | 0.8 | 0 | 0 |
| Active schools coordinators (*n*=6) | 0.25 | 0.25 | 0.5 | 0.25 | **1.5** | 0.25 | 0 |
| Headteachers (*n*=6) | 0.3 | 0.2 | 0.3 | **1.0** | 0.7 | 0.2 | 0.3 |
| Teachers (*n*=6) | 0.5 | 0 | 0 | **1.3** | 0.8 | 0.2 | 0.2 |
| Active partner school specialists (*n*=6) | 0.6 | 0.2 | 0.5 | **1.0** | 0.5 | 0.2 | 0.0 |
| National organisation representatives (*n*=5) | **1.0** | 0.0 | 0.6 | 0.6 | 0.4 | 0.2 | 0.2 |
| Local delivery pilot representatives (*n*=5) | 0.0 | 0.0 | 0.0 | **2.0** | 0.0 | 1.0 | 0.0 |
| International researchers (*n*=5) | 0.4 | 0.0 | 0.0 | **1.6** | 0.8 | 0.2 | 0.0 |
| Overall (*n*=50) | 0.5 | 0.1 | 0.2 | **1.2** | 0.7 | 0.2 | 0.1 |

**Bold text** indicates the framework with the most votes by stakeholder group, and overall.
